# Astroglial NF-kB contributes to white matter damage and cognitive impairment in a mouse model of vascular dementia

**DOI:** 10.1186/s40478-016-0350-3

**Published:** 2016-08-04

**Authors:** Raman Saggu, Toni Schumacher, Florian Gerich, Cordula Rakers, Khalid Tai, Andrea Delekate, Gabor C. Petzold

**Affiliations:** 1 German Center for Neurodegenerative Diseases (DZNE), Ludwig-Erhard-Allee 2, 53175 Bonn, Germany; 2 Department of Neurology, University Hospital Bonn, Sigmund-Freud-Str. 25, 53127 Bonn, Germany

**Keywords:** Astrocytes, vascular cognitive impairment, Glia, NF-kB, Diffusion tensor imaging (DTI), Neuroinflammation

## Abstract

Vascular cognitive impairment is the second most common form of dementia. The pathogenic pathways leading to vascular cognitive impairment remain unclear but clinical and experimental data have shown that chronic reactive astrogliosis occurs within white matter lesions, indicating that a sustained pro-inflammatory environment affecting the white matter may contribute towards disease progression. To model vascular cognitive impairment, we induced prolonged mild cerebral hypoperfusion in mice by bilateral common carotid artery stenosis. This chronic hypoperfusion resulted in reactive gliosis of astrocytes and microglia within white matter tracts, demyelination and axonal degeneration, consecutive spatial memory deficits, and loss of white matter integrity, as measured by ultra high-field magnetic resonance diffusion tensor imaging. White matter astrogliosis was accompanied by activation of the pro-inflammatory transcription factor nuclear factor (NF)-kB in reactive astrocytes. Using mice expressing a dominant negative inhibitor of NF-kB under the control of the astrocyte-specific glial fibrillary acid protein (GFAP) promoter (GFAP-IkBα-dn), we found that transgenic inhibition of astroglial NF-kB signaling ameliorated gliosis and axonal loss, maintained white matter structural integrity, and preserved memory function. Collectively, our results imply that pro-inflammatory changes in white matter astrocytes may represent an important detrimental component in the pathogenesis of vascular cognitive impairment, and that targeting these pathways may lead to novel therapeutic strategies.

**Electronic supplementary material:**

The online version of this article (doi:10.1186/s40478-016-0350-3) contains supplementary material, which is available to authorized users.

## Introduction

Vascular cognitive impairment (VCI) is a common neurodegenerative disease characterised by impaired cognitive function attributable to cerebrovascular pathology [1]. In its pure form, VCI is the second most common form of dementia and represents an important co-factor for the development of Alzheimer’s disease and other neurodegenerative disorders [2]. Neuropathologically, VCI is characterised by demyelination and axonal loss within white matter tracts [3], which has traditionally been attributed to the direct effects of chronic oxygen deprivation. However, several lines of evidence suggest that chronic neuroinflammation may also play a detrimental role in the initiation and progression of VCI. First, cerebrovascular and cardiovascular diseases, which are risk factors for VCI, induce a chronic pro-inflammatory environment, particularly in and around blood vessels [4, 5]. Second, increased levels of pro-inflammatory markers have been detected within white matter lesions in animal models and post-mortem studies of VCI patients [6, 7], and alterations of blood–brain barrier integrity are a central and perhaps aggravating element of VCI [8, 9]. Finally, in patients and animal models of VCI, reactive gliosis of microglia and astrocytes is present within the white matter [10, 11]. Reactive astrogliosis is a pervasive defence mechanism in response to cerebral injury that may promote neurodegeneration or neuroprotection in a context-dependent manner [12]. However, the roles of astrogliosis and the various inflammatory cascades mediated by reactive astrocytes in chronic cerebral hypoperfusion and VCI remain poorly understood.

Here, we have used an animal model that recapitulates many of the neuropathological and behavioural hallmarks of VCI [13]. We demonstrate that reactive astrogliosis induces activation of the pro-inflammatory nuclear factor (NF)-kB pathway in astrocytes, and that transgenic inhibition of this pathway confers protection against axonal loss, helps to promote white matter integrity, and preserves cognitive function.

## Materials and methods

### Animals

All experiments were approved by a local animal welfare committee. We used 10–14 week-old male GFAP-IkBα-dn mice [14] and their wildtype age-matched male littermates. Animals were housed in groups (two to five per cage) on a 12 h light/dark cycle with food and water available *ad libitum*.

### Chronic hypoperfusion model

Gold plated stainless steel microcoils (Sawane) were wrapped around both common carotid arteries in isoflurane-anaesthetised mice [13]. Informed by earlier studies that revealed either subtle [15, 16] or very pronounced changes [17], we modified the existing model by using custom-made coils with an internal diameter of 170 μm. Animals were anaesthetised with isoflurane (1.5 % v/v) and maintained on a heating pad (37 °C). The common carotid arteries were exposed through a midline cervical incision, and the microcoils were wrapped around both common carotid arteries. A 30 min interval was allowed between each microcoil insertion. Sham-treated animals underwent identical procedures except that coils were not implanted. Local lidocaine gel and intraperitoneal buprenorphine were applied as analgesics. Mice from each litter were randomised to either microcoil insertion or sham surgery. All experiments and subsequent data analysis were conducted in a blinded manner, with respect to genotype and treatment. Mice were excluded if visual damage to the carotid arteries or bleeding during surgery occurred. In addition, carotid arteries were inspected for patency at the end of experiments, and animals with occluded carotid arteries were also excluded.

### Laser speckle perfusion imaging and PcomA assessment

Animals were anaesthetised with isoflurane (1.5 % v/v), and the skull was exposed through a midline incision. A cranial window was created and closed by gluing a glass coverslip onto the skull and adhering the skin to the coverslip rim. For imaging, mice were anaesthetised with isoflurane (1 % v/v) and fixed within a stereotaxic frame (Narishige). Brain perfusion was determined through a closed cranial window in anaesthetised mice using PeriCam PSI Laser Speckle Imager and PIMsoft software (Perimed). PcomA development was determined by transcardial perfusion with India ink (Pelikan; 10 % in water containing 10 % gelatine), followed by decapitation and fixation in 4 % paraformaldehype (PFA). PcomA was scored as: 0, absent; 1, hypoplastic; 2, truncal. A single PcomA score was calculated by averaging left and right scores.

### Ultra high-field MRI

MRI of isoflurane-anaesthetised mice was performed on an 11.7 T horizontal small-bore magnet (Biospec 117/16, Bruker) using a two-element transmit/receive proton (^1^H) cryocoil (Bruker Biospin). Body temperature was maintained at 37 °C via an integrated water heating system. Respiratory rate was maintained at 70-80 cycles/min. Anatomical images were acquired using a rapid acquisition relaxation enhancement (RARE) T_2_-weighted (T_2_-w) sequence [echo time (TE) = 0.01 s; repetition time (TR) = 2.5 s; in-plane resolution 45 μm^2^], and a RARE T_1_-weighted (T_1_-w) sequence (TE/TR = 0.01 s/1 s; in-plane resolution 45 μm^2^). White matter integrity was assessed using an EPI DTI sequence (TE/TR 26/4000 ms; *b* value 1000 s/mm^2^; 60 diffusion directions; 5 b0 images; in-plane resolution 100 μm^2^).

### Immunohistochemistry

Coronal sections (20 μm) were obtained from PFA-fixed hemispheres, blocked with 10 % normal goat serum (Vector Labs) and 0.3 % Triton-X100 (Sigma) in PBS for 1 h, and incubated with primary antibodies in PBS containing 5 % goat serum and 0.05 % Triton overnight at 4 °C. The following primary antibodies were used: rabbit anti-GFAP (1:1000; Z0334, Dako), rat anti-GFAP (1:1000; 13-0300, Invitrogen), rabbit anti-Iba1 (1:500; #019-19741, Wako), mouse anti-SMI312 (1:500, smi-312r, Covance), rabbit anti-p65 (1:100; sc-372, Santa Cruz; or #3033, Cell Signaling). Stainings were visualised using secondary antibodies from goat conjugated with Alexa Fluor 488, 596 or 647 (1:1000; Invitrogen; 2 h at room temperature). Nuclei were stained with Hoechst 33258 (1:1000; Invitrogen). Omission of primary antibodies served as negative controls. Confocal images were acquired using a confocal laser scanning microscope (LSM 700; Zeiss).

### Cytokine and NF-kB quantification

Mice were sacrificed, and the corpus callosum was dissected and stored in liquid nitrogen. Samples were homogenised in PBS containing protease/phosphatase inhibitor (Thermo Scientific) using a Precellys 24 homogeniser (Peqlab), and lysed in RIPA buffer (25 mM TRIS, 150 mM NaCl, 1 % NP-40, 0.5 % Na-deoxycholate, 0.1 % SDS, pH 7.2). Quantitative cytokine determination was performed using an electrochemiluminescence ELISA (Mouse ProInflammatory Ultra-Sensitive Kit, Meso Scale Discovery). Signals were measured on a SECTOR Imager 2400 reader (Meso Scale Discovery). For NF-kB measurements, nuclear extracts were created from whole brain homogenates (Nuclear Extraction Kit, #2900, Millipore), and NF-kB activity in nuclear extracts was determined with a commercial assay (EZ-TFA assay, #70-610, Millipore) according to the manufacturer’s instructions using a microplate reader (FLUOstar Omega, BMG Labtech). All measurements were performed in duplicates.

### Behavioural tests

Male GFAP-IkBα-dn and wildtype mice have similar phenotypes in behavioural paradigms, including spatial learning and memory [18]. All tests were recorded and analysed using Ethovision XT9 (Noldus). The Y maze consisted of three arms (length, 34.5 cm; width, 8 cm; height, 12.5 cm) extruding at equal angles from a central platform. The Y Maze test was modified from previous designs [19]. During the acquisition trial, one arm was closed and mice were placed at the end of an open arm (chosen at random) and allowed to explore the two open arms for 5 min. After 24 h, mice were returned to the maze with all 3 arms open; the start point corresponding to the individual start point of each mouse in the acquisition trial, and allowed to explore for 5 min. Arm preference was quantified as the ratio between the time spent in one arm and the time spent in all arms (preferency index). For open field exploration, mice were placed in the centre of a dimly lit (20–30 lx) arena. The area was virtually divided into a centre and an area ≤5 cm to the walls (surround). General locomotor activity was quantified as total distance moved and velocity. The level of anxiety was estimated by the percentage of time spent in the surround.

### Data analysis

Immunohistochemical images were imported into ImageJ 1.50 (W. Rasband, NIH), smoothed and despeckled using a median filter, and converted to an 8-bit grey level image. Following contrast enhancement (0.4 % saturated pixels), images were binarised with a threshold defined as the mean background intensity plus the standard deviation of background intensity multiplied by 3. Astrocyte area coverage and diameter, and micoglial cell count were determined automatically by ImageJ. For astrocyte process length, binarised images were skeletonised and analysed using the Analyze Skeleton (2D/3D) plugin for ImageJ. These semi-automatic quantifications were compared with a subset of data (*n =* 3 from each group) that was quantified by manual analysis; no significant difference was detected between the analysis methods (data not shown). GFAP-positive astrocytes were considered positive for p65 when they showed a spherical somatic p65 staining that was smaller than the GFAP-positive cell soma and overlapped with the Hoechst 33258 nuclear counterstain. Mean pixel intensity of SMI312 staining in 8-bit grey level images was taken as a measure of axonal integrity. Demyelination was analysed in LFB-stained sections using a semi-quantitative scoring system (0 = no demyelination; 1 = visible fiber disarrangement; 2 = vacuoles and focal demyelination; 3 = widespread/continuous demyelination). Examples are provided in Additional file 1: Figure S1. Analysis of the corpus callosum was performed within pre-defined areas (0.75 mm and 2.0 mm from the midline). All measurements were taken in triplicates, and the values averaged. Laser speckle contrast data were down-sampled, exported into Excel (Microsoft), and normalised to perfusion levels before microcoil implantation. Paravision 5.1 (Bruker) was used to generate the parametric images used for white matter analysis. Fractional anisotropy (FA), apparent diffusion coefficient (ADC), axial diffusivity (AD) and radial diffusivity (RD) were measured. Direction-encoded colour (DEC) maps based on the primary eigenvectors were generated using in-house software. The colour determines the principal direction (X, Y or Z) of the primary eigenvector exhibiting the largest diffusion coefficient from the measured diffusion tensor: red designates medial to lateral orientation, blue represents dorsal to ventral orientation and green exhibits the rostral to caudal orientation. Regions of interest (ROIs) including the corpus callosum, external capsule and internal capsule were manually drawn onto the diffusion tensor images, and verified by comparison with the corresponding T_2_-w image and a stereotaxic mouse brain atlas [20]. Heatmaps were generated by a) registering the FA images to a reference image within the respective cohort and b) setting landmarks on trace-weighted diffusion images and transferring them onto their respective FA images. Registration was performed using the bUnwarp plugin (version 2.6.3) for ImageJ.

### Statistical analysis

Comparisons between two groups were conducted using the Mann–Whitney test. Differences between several groups were analysed using the Kruskal-Wallis test followed by Dunn’s multiple comparisons test. Data were analysed using Prism 6 (GraphPad), and are expressed as mean ± SEM; *p <* 0.05 was accepted as statistically significant.

## Results

### Gliosis, demyelination, axonal loss and memory deficits in a mouse model of VCI

**Fig. 1:**
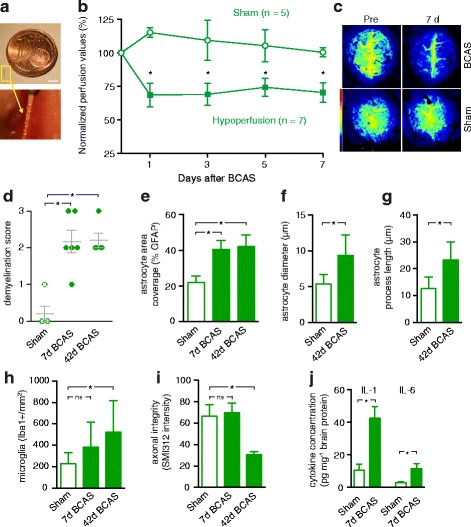
Chronic hyperperfusion induces demyelination, gliosis, axonal deterioration, and cognitive impairment. **a** Mice were subjected to bilateral carotid artery stenosis (BCAS) by wrapping gold-plated microcoils (internal diameter, 170 μm) around the common carotid arteries. Scale bar, 3 mm. **b** Laser speckle contrast imaging of anaesthetised mice revealed long-lasting cerebral hypoperfusion after BCAS compared to sham-treated mice (*p <* 0.05, Mann–Whitney test). **c** Representative examples of laser speckle contrast measurements of cerebral blood flow through chronic cranial windows in mice subjected to BCAS or sham surgery before (Pre) and 7 d after the intervention. The look-up table represents arbitrary perfusion units, indicating reduced blood flow after BCAS. **d** Luxol Fast Blue staining of the corpus callosum (0 = no demyelination; 1 = visible fiber disarrangement; 2 = vacuoles and focal demyelination; 3 = widespread/continuous demyelination) revealed demyelination 7 d and 42 d after BCAS (*n =* 6 for each group; *p <* 0.05, Kruskal-Wallis test followed by Dunn’s multiple comparisons test). **e**-**g** The area covered by GFAP-positive astrocytes, astrocyte diameter and astrocyte process length were increased in mice subjected to BCAS in the corpus callosum, indicating reactive astrogliosis (*n =* 6 for each group; *p <* 0.05, Mann–Whitney test). **h** Mild reactive microgliosis was also evident 6 weeks after BCAS, indicated by an increase in Iba1-positive cells (*n =* 6 for each group; *p <* 0.05, Mann–Whitney test). **i** In parallel, there was a decrease in the density of SMI312-positive axons in the corpus callosum after 6 weeks, indicating axonal loss (*n =* 6 for each group; *p <* 0.05, Mann–Whitney test). **j** Electrochemiluminescence measurements revealed an increase in the pro-inflammatory cytokines IL-1 and IL-6 in homogenates from the corpus callosum (*n =* 8 for each group; *p <* 0.05, Mann–Whitney test)

We modified a model of bilateral common carotid artery stenosis (BCAS) [13] by placing microcoils around both common carotid arteries to induce chronic cerebral hypoperfusion in mice (Fig. 1a). Longitudinal laser speckle imaging demonstrated a reduction in cerebral blood flow by 32 ± 9 % 24 h and 29 ± 8 % 7 days following BCAS induction, respectively, whereas blood flow remained unaltered in sham-treated mice (Fig. 1b-c). Separate cohorts of mice, perfused either 1 week or 6 weeks after BCAS induction, exhibited white matter demyelination (Fig. 1d), reactive astrogliosis (Fig. 1e-g), microglial activation (Fig. 1h), and delayed degradation of white matter axonal integrity (Fig. 1i). Reactive astrogliosis was evident by increased area coverage, cell size and process length, although the latter parameter is also influenced by GFAP redistribution induced by astrogliosis [12]. The number of GFAP-positive astrocytes in the corpus callosum did not differ between the groups, owing to the high fraction of GFAP-positive astrocytes already present in the white matter (148 ± 12/mm^2^ and 159 ± 10/mm^2^, respectively; *n =* 6 for each group; *p >* 0.05, Mann–Whitney test). No difference was seen in the number of NeuN-positive neuronal cell bodies in the cortex (Sham, 1987 ± 211/mm^2^; BCAS, 2027 ± 274; *n =* 6 for each group; *p >* 0.05, Mann–Whitney test). There was no change in the rostro-caudal diameter of the corpus callosum, measured in coronal sections, in mice subjected to BCAS compared with sham-treated mice (211 ± 26 μm vs. 201 ± 32 μm; *n =* 6 for each group; *p >* 0.05, Mann–Whitney test). Representative examples are shown in the Additional file 2: Figure S2. Changes comparable to those observed in the corpus callosum were detected in the internal capsule and external capsule (Additional file 3: Figure S3).

Moreover, we found that the concentrations of the pro-inflammatory cytokines, interleukin-1 and interleukin-6, were increased in homogenates of the corpus callosum isolated from mice subjected to BCAS (Fig. 1j).

**Fig. 2:**
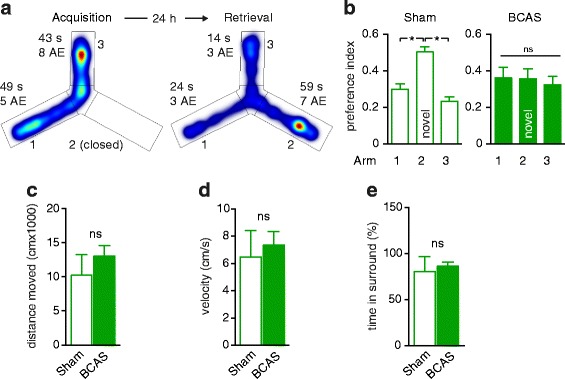
Chronic hypoperfusion induces a decline in spatial long-term memory. **a** Representative example of spatial memory determination in the Y Maze test. During the acquisition trial, arm 2 is closed, and the mouse moves freely between arms 1 and 3. 24 h later, in the retrieval trial, arm 2 is opened. The mouse showed a strong preference for this novel arm, indicated by longer cumulative duration and more arm entries (AE). **b** Sham-treated mice spent significantly more time in the novel arm (*n =* 12; *p <* 0.05, Kruskal-Wallis test followed by Dunn’s multiple comparisons test) whereas mice subjected to BCAS spent equal times in all arms, indicating spatial memory impairment (*n =* 12; *p >* 0.05, Kruskal-Wallis test followed by Dunn’s multiple comparisons test). **c**-**e** The distance travelled and velocity of wildtype mice subjected to sham surgery or BCAS did not differ significantly. The percentage of time spent in the surround (i. e. ≤5 cm to the wall of the arena), indicative of the level of anxiety, was also similar (*n =* 12 for each group; Mann–Whitney test)

To investigate spatial long-term memory, we used a two-trial Y maze spatial memory task [19]. In the first trial (acquisition), mice were allowed to visit two arms of the maze, while the third arm was blocked. In the second trial (retrieval) performed 24 h later, mice had access to all three arms. As expected, given the natural tendency of rodents to explore new environments, sham-treated mice showed a strong preference for the novel arm in the retrieval trial (Fig. 2a-b). By contrast, mice subjected to BCAS showed no preference for the novel arm (Fig. 2b), indicating decreased spatial memory function. Spontaneous locomotor activity and the level of anxiety, which were investigated in the open field test, did not differ between the groups (Fig. 2c-e) indicating that the deficits of hypoperfused mice in the Y maze test were due to impaired memory function and not to a decline in motor function or general behavioural alterations.

### NF-kB is activated by chronic hypoperfusion in reactive white matter astrocytes

One consequence of the expression of pro-inflammatory cytokines such as IL-1 is the activation of the nuclear factor (NF)-kB signaling cascade in reactive astrocytes [21]. Under control conditions, inactive NF-kB is localised to the cytoplasm by its interaction with inhibitory IkB proteins such as IkBα. Dissociation from IkB and subsequent nuclear translocation of NF-kB is initiated by the degradation of IkB through cytokine-induced IkB kinase activation [22]. Once activated, NF-kB acts as an important regulator of reactive gliosis, inflammation, myelination and neuronal survival in different disease models [12, 14, 23].

**Fig. 3:**
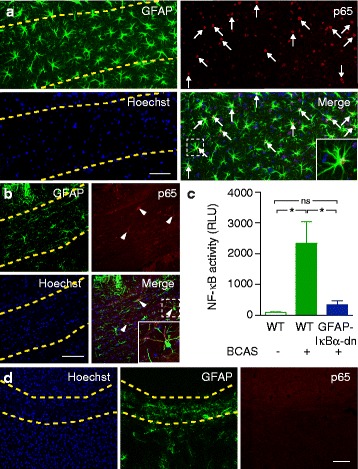
Activation of the NF-kB pathway in reactive white matter astrocytes during chronic hypoperfusion. **a** Reactive GFAP-positive astrocytes (*green*) in the corpus callosum of mice subjected to BCAS are positive for the active p65 (RelA) subunit of NF-kB (*red*). p65 was localised to astroglial nuclei (*arrows*), indicated by co-localisation with the nuclear marker Hoechst 33258 (*blue*). **b** Astrocytes were rarely positive for p65 in sham-treated mice, and the signal was diffuse and localised to the cytosol (arrowheads), indicating non-activity. **c** Biochemical determination of NF-kB activity in brain homogenates showed strong activation of the NF-kB pathway in wildtype mice subjected to BCAS but not in GFAP-IkBα-dn mice subjected to BCAS or sham-treated wildtype mice (*n =* 6 for each group; Kruskal-Wallis test followed by Dunn’s multiple comparisons test). **d** Within the corpus callosum of GFAP-IkBα-dn mice subjected to BCAS, immunohistochemistry for active p65 was also negative. Scale bars, 100 μm. Insets in A-B correspond to dashed squares and are 80 μm in width. Yellow dashed lines indicate location of the corpus callosum

Therefore, we investigated the occurrence of activated nuclear NF-kB by immunohistochemistry against the active p65 (RelA) subunit of NF-kB. We found that 27.2 ± 7.1 % of all GFAP-positive white matter reactive astrocytes in hypoperfused mice were positive for nuclear (i.e. active) p65 (*n =* 7 mice; Fig. 3a). By contrast, only sparse and cytosolic (i.e. inactive) p65 labeling was visible in astrocytes from sham-treated mice (3.8 ± 1.8 %, *n =* 6 mice; Fig. 3b). White matter microglia and neurons were only very rarely positive for nuclear p65 in chronic hypoxic mice (3.4 ± 0.9 % of Iba1-positive microglia and 0.8 ± 0.3 % of NeuN-positive neurons, respectively). Hoechst-positive and GFAP-negative punctuate p65 signals were rare (7.3 ± 2.4 % of all Hoechst-positive p65 signals). These data indicate that the majority of white matter NF-kB activation in our model occurs in astrocytes. To quantify NF-kB activity, we used a chemiluminescent assay measuring the amount of p65 bound to the flanked DNA binding consensus sequence for NF-kB in nuclear extracts from brain homogenates. Consistent with the immunohistochemistry, this assay demonstrated strong NF-kB activation in the brains of mice subjected to BCAS compared with sham (Fig. 3c). These data indicate that reactive astrogliosis and chronic inflammation of the white matter in hypoperfused mice induces NF-kB activation in reactive astrocytes.

### Astrocyte-specific transgenic inhibition of NF-kB activity confers protection against structural and cognitive decline following chronic hypoperfusion

To investigate the consequences of astroglial NF-kB activation, we used transgenic GFAP-IkBα-dn mice in which astroglial NF-kB activation is selectively inhibited by targeting the overexpression of a dominant negative form of IkBα to astrocytes [14]. Earlier studies have shown that the transgene is selectively active in astrocytes, while NF-kB activity remains unchanged in neurons [14]. Following BCAS, we found that nuclear p65 was absent from reactive white matter astrocytes (Fig. 3d) and that NF-kB activity was similar to sham-treated mice (Fig. 3c).

**Fig. 4:**
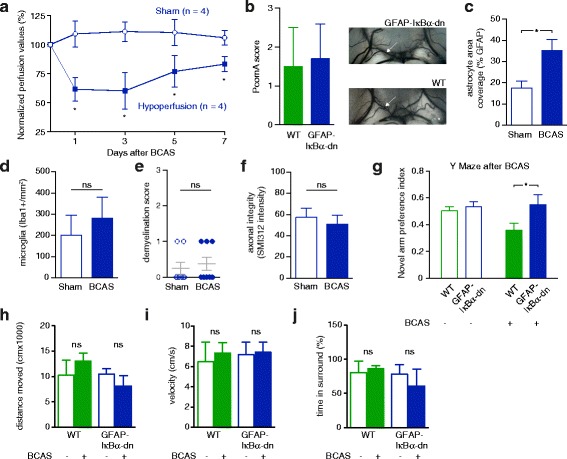
GFAP-IkBα-dn mice are protected from the deleterious effects of chronic hypoperfusion. **a** Laser speckle contrast imaging of GFAP-IkBα-dn mice demonstrated cerebral hypoperfusion after BCAS compared with sham-treated mice (*p <* 0.05, Mann–Whitney test) similar to blood flow levels observed in wildtype mice shown in Fig. 1. **b** India ink angiograms revealed that the patency/development of the posterior communicating artery (PComA) was similar in wildtype and GFAP-IkBα-dn mice (*n =* 5 for each group; *p >* 0.05, Mann–Whitney test). **c**-**f** Reactive astrogliosis was evident in GFAP-IkBα-dn mice after BCAS. However, microgliosis, demyelination and loss of axonal integrity were strongly ameliorated (6 weeks after BCAS; *n =* 6 for each group and staining; Mann–Whitney test for each comparison). **g** 6 weeks after BCAS, GFAP-IkBα-dn mice spent significantly more time in the novel arm during the retrieval trial of the Y maze compared to wildtype mice subjected to BCAS, indicating preserved spatial memory function (*n =* 12 for each group; Mann–Whitney test; Two-way ANOVA: *F*(1, 46) = 5.52, *p =* 0.02 for genotype effect). **h**-**j** The distance travelled and velocity, as well as the level of anxiety, of GFAP-IkBα-dn mice subjected to sham surgery or BCAS did not differ significantly and was similar to wildtype mice. Wildtype mouse data in G-J correspond to those in Fig. 2

The level and time course of cerebral blood flow reduction (Fig. 4a) and the plasticity of the posterior communicating artery (PcomA; Fig. 4b) were similar in GFAP-IkBα-dn and wildtype mice (Laser speckle blood flow levels from each individual day of measurement were compared between GFAP-IkBα-dn and wildtype mice; *p >* 0.05 for each comparison, Mann–Whitney test). Thus, differences between these groups are unlikely to be due to variability in the degree of hypoperfusion or collateralisation between the anterior and posterior cerebral artery circulation.

Interestingly, astrogliosis was detectable in GFAP-IkBα-dn mice following BCAS but reactive microglial activation and demyelination in white matter structures were significantly attenuated (Fig. 4c-e). Furthermore, axonal integrity within the corpus callosum was preserved in GFAP-IkBα-dn mice compared with sham-treated controls (Fig. 4f). Importantly, GFAP-IkBα-dn mice subjected to hypoperfusion spent significantly more time in the novel arm in the retrieval trial of the Y maze test (Fig. 4g), indicating preserved spatial memory function as a consequence of decreased NF-kB signaling. The open field test revealed no differences in distance moved, velocity or time spent in the surround between hypoperfused GFAP-IkBα-dn, sham-treated GFAP-IkBα-dn and wildtype mice (Fig. 4h-j). Overall, these data show that NF-kB activation in reactive astrocytes contributes to neuropathological damage and behavioural changes in our VaD model.

### Ultra high-field diffusion tensor imaging reveals preserved structural integrity of white matter tracts in GFAP-IkBα-dn mice following BCAS

**Fig. 5:**
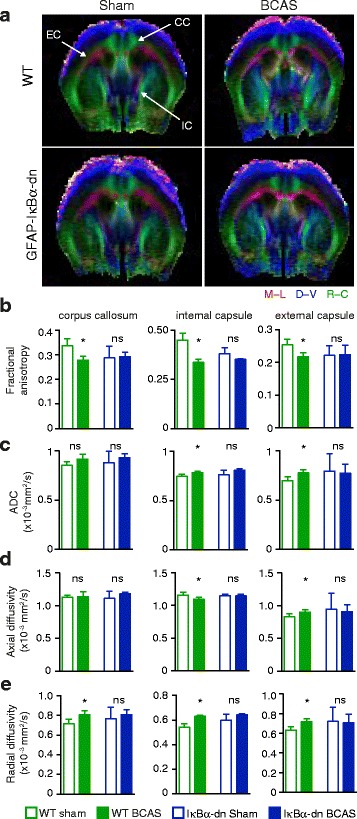
Ultra high-field diffusion tensor imaging reveals preserved structural integrity of major white matter tracts in GFAP-IkBα-dn mice. **a** Anaesthetised wildtype or GFAP-IkBα-dn mice, either sham-treated or subjected to BCAS, were imaged using ultra high-field DTI MRI. Major white matter tracts (CC, corpus callosum; IC, internal capsule; EC, external capsule) were identified in direction-encoded colour (DEC) images (red, medial to lateral orientation; blue, dorsal to ventral orientation; green, rostral to caudal orientation) based on the primary eigenvectors. **b** Fractional anisotropy was significantly decreased in all regions of interest in wildtype but not in GFAP-IkBα-dn mice subjected to BCAS. **c** BCAS induced significant changes in the apparent diffusion coefficient (ADC) of the internal capsule and external capsule in wildtype but not in GFAP-IkBα-dn mice. **d**-**e** Axial and radial diffusivity were also significantly altered in wildtype but not in GFAP-IkBα-dn mice subjected to BCAS (wildtype sham, *n =* 6; wildtype BCAS, *n =* 5; GFAP-IkBα-dn sham, *n =* 3; GFAP-IkBα-dn BCAS, *n =* 5; Mann–Whitney test for all comparisons)

Magnetic resonance diffusion tensor imaging (DTI) is a key technology that enables the non-invasive study of structural connectivity of white matter tracts, and is an invaluable tool for the diagnosis and longitudinal study of patients with vascular dementia [24, 25]. Therefore, we investigated whether the preservation of axonal integrity observed in GFAP-IkBα-dn mice was detectable by DTI MRI. DTI was performed on hypoperfused or sham-treated wildtype and GFAP-IkBα-dn mice 6 weeks post surgery. Coronal images from brain areas encompassing the major white matter tracts (corpus callosum, internal capsule and external capsule) were acquired, and fractional anisotropy (FA), apparent diffusion coefficient (ADC), axial (AD) and radial diffusivity (RD) were measured. Representative direction-encoded colour (DEC) maps are shown in Fig. 5a. We detected significant changes in white matter FA, ADC, AD and RD values in wildtype mice subjected to BCAS compared with sham-treated mice (Fig. 5b-e and Additional file 4: Figure S4). This indicates that hypoperfusion induced deterioration in white matter integrity consistent with the observed histopathological changes. By contrast, GFAP-IkBα-dn mice subjected to BCAS mice did not exhibit a difference in the diffusivity measures when compared with sham-treated GFAP-IkBα-dn mice, underlining the strong attenuation in pathology compared with wildtype animals (Fig. 5b-e). T_1_- and T_2_-weighted imaging revealed no signs of overt anatomical damage in any group (Additional file 5: Figure S5).

## Discussion

In this study, we investigated the role of the pro-inflammatory NF-kB pathway in reactive astroglia in a mouse model of vascular cognitive impairment and found that transgenic inhibition of NF-kB signaling confers protection from axonal loss, gliosis, demyelination, deterioration of white matter integrity and memory impairment.

Although vascular cognitive impairment is associated with an enormous clinical and socioeconomic burden, causal treatment options are absent, mostly because the molecular pathways triggered by chronic cerebral hypoperfusion remain poorly understood. Importantly, clinical and experimental studies have shown that vascular impairment is associated with inflammatory changes and reactive gliosis of the white matter [4–7, 9], indicating that white matter damage is not simply a consequence of chronic oxygen deprivation but is induced and sustained by a pro-inflammatory environment. The hallmarks of chronic inflammation:reactive gliosis of white matter astrocytes and microglia, and expression of pro-inflammatory cytokines, were also detected in our model and associated with axonal degeneration, demyelination, spatial memory impairment and loss of white matter integrity. Importantly, the DTI signatures of hypoperfused mice corroborated with the demyelination and axonal degeneration observed on histology [26], and were comparable to those observed in clinical vascular cognitive impairment [27]. The cellular sources of IL-1 and IL-6 are difficult to define *in vivo* using our model. However, earlier studies have shown that microglia and, to a lesser degree, astrocytes release these cytokines during hypoxia [1, 23, 28]. Hence, both cell types may induce and sustain the pro-inflammatory environment that ultimately leads to demyelination. Moreover, progressive demyelination may further exacerbate gliosis [28].

Reactive astrogliosis is a pervasive but insufficiently understood response mechanism of the brain to acute or chronic injury [12]. Interestingly, we found that astrogliosis induces activation of the pro-inflammatory NF-kB pathway, and that transgenic inhibition of NF-kB signaling in reactive astrocytes confers protection from neurodegeneration and cognitive impairment. Notably, some degree of gliosis was evident in transgenic mice after BCAS, indicating that multiple pathways may be involved [12]. The protective effect of NF-kB on white matter integrity was also detectable using DTI, underlining the significance of DTI as a longitudinal marker of treatment response in future translational studies.

It should be noted that the model used in the present study, and arguably in previously published animal studies thus far [11], does not reflect certain aspects of clinical vascular cognitive impairment including the chronic evolution of the disease over decades, the predominant affection of small vessels, and cardiovascular and environmental co-morbidities. Therefore, an important question for future studies is whether the protective effect of NF-kB inhibition is evident in models that recapitulate other aspects of human vascular cognitive impairment. One other important point to address will be whether astroglial NF-kB contributes to pathways such as those mediated by hypoxia-inducible transcription factor-1 activation or perturbed glutamate uptake [29, 30] in models of vascular cognitive impairment. Furthermore, as NF-kB pathway inhibitors progress through pre-clinical development [31], it will be of great interest to investigate whether these compounds confer comparable protection from chronic hypoxia.

The NF-kB pathway has been implicated in other pathological conditions such as autoimmune or toxic demyelination and spinal cord injury [14, 23, 32]. Here, we demonstrated that activation of NF-kB in astrocytes contributes not only to white matter demyelination and axonal loss during chronic hypoxia but that an astrocytic pro-inflammatory pathway may have important consequences for cognitive outcome in neurodegenerative disease.

## Conclusions

Our study has demonstrated that chronic activation and gliosis of astrocytes are central components of disease pathogenesis in a model of vascular cognitive impairment, and that therapeutic strategies targeting these pathways may lead to novel treatment options for vascular dementia.

## Abbreviations

DTI, Diffusion tensor imaging; GFAP, Glial fibrillary acid protein; MRI, Magnetic resonance imaging; NF-kB, Nuclear factor kappa-light-chain-enhancer of activated B cells; VCI, Vascular cognitive impairment

## Additional file

Additional file 1: Figure S1. Scoring system to measure demyelination. Representative LFB stainings from the corpus callosum and the corresponding grades are provided. (PDF 2173 kb) Additional file 2: Figure S2. Histological and immunohistochemical changes induced by BCAS. The corpus callosum is delineated by yellow dashed lines in all images. (A) Luxol Fast Blue (LFB) staining of the corpus callosum in sham-treated mice and mice subjected to BCAS revealed fibre disarrangement, vacuoles and focal demyelination following BCAS. (B) Astrocytes, stained by an anti-GFAP antibody, became reactive, as indicated by higher area coverage, larger cell diameters, and longer processes. (C) The number of Iba1-positive microglia increased within the corpus callosum. (D) The signal intensity of axons stained with the pan-axonal marker SMI312 decreased after BCAS, indicative of axonal damage. (E) The number of NeuN-positive neuronal cell bodies was comparable in both groups. Scale bars, 100 μm. (PDF 7114 kb) Additional file 3: Figure S3. Histological changes in the internal and external capsule. Reactive astrogliosis (A, E), microgliosis (B, F), axonal degeneration (C, G) and demyelination (D, H) in sham and BCAS animals in the internal capsule (upper row) and external capsule (lower row). (PDF 118 kb) Additional file 4: Figure S4. Fractional anisotropy maps. Representative examples of fractional anisotropy maps in two coronal imaging planes (relative to Bregma) in wildtype or GFAP-IkBα-dn mice subjected to BCAS or sham surgery, respectively. (PDF 6689 kb) Additional file 5: Figure S5. T_1_- and T_2_-weighted images show no changes following BCAS. Representative examples of T_1_- and T_2_-weighted coronal images in wildtype or GFAP-IkBα-dn mice subjected to BCAS or sham surgery, respectively. These images show that grey and white matter structures are devoid of pathological signal changes. (PDF 2360 kb)
