# Supplementary figures and images for: Astroglial NF-kB contributes to white matter damage and cognitive impairment in a mouse model of vascular dementia
Source: Acta Neuropathol Commun. 2016 Aug 4;4:76. doi: 10.1186/s40478-016-0350-3 (PMC4973061; doi:10.1186/s40478-016-0350-3)

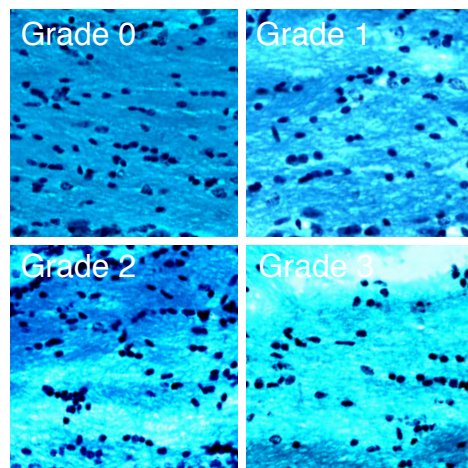

Supplement: Additional file 1: Figure S1. — Scoring system to measure demyelination. Representative LFB stainings from the corpus callosum and the corresponding grades are provided. (PDF 2173 kb) [file 40478_2016_350_MOESM1_ESM.pdf]

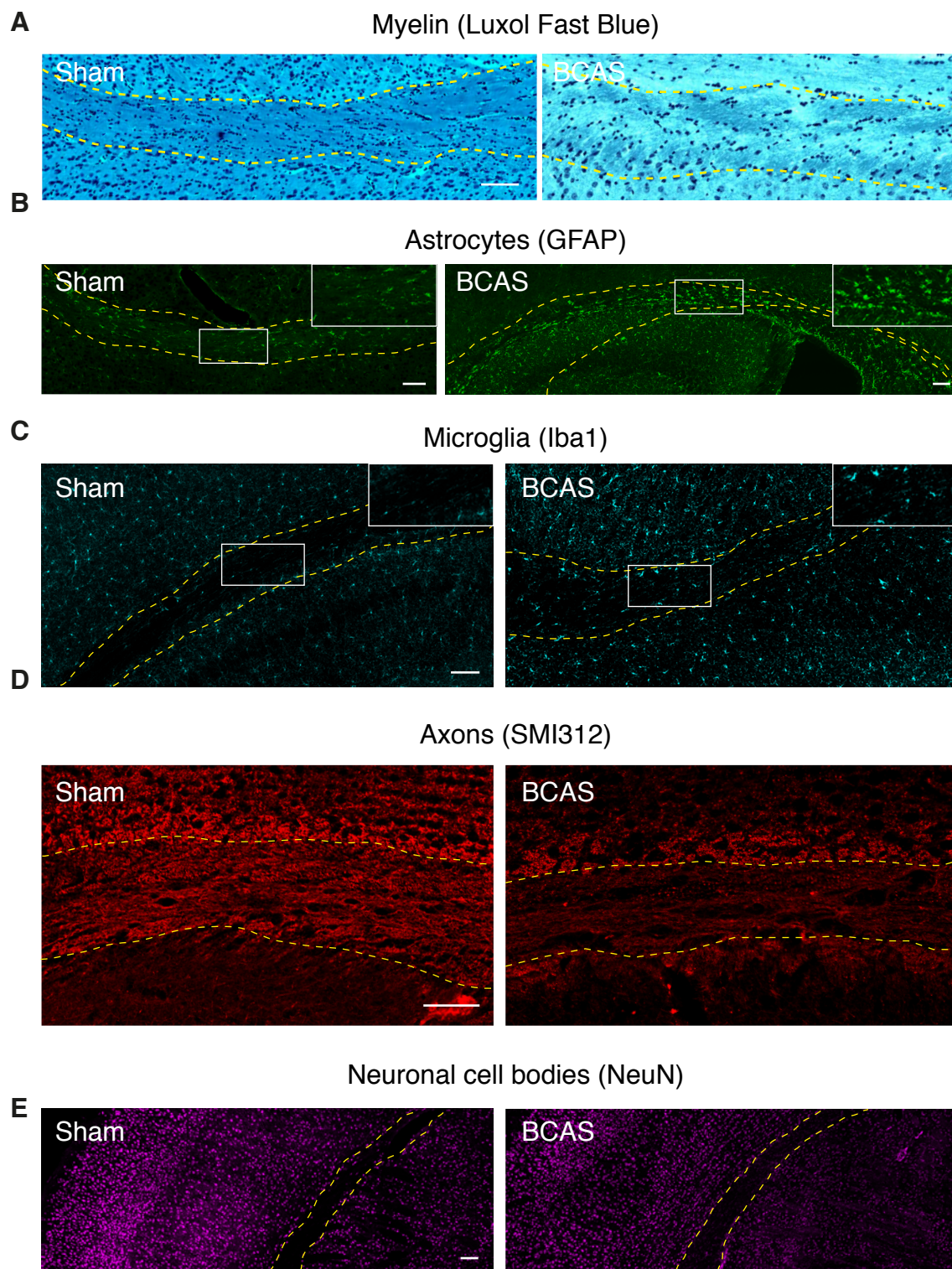

Supplement: Additional file 2: Figure S2. — Histological and immunohistochemical changes induced by BCAS. The corpus callosum is delineated by yellow dashed lines in all images. (A) Luxol Fast Blue (LFB) staining of the corpus callosum in sham-treated mice and mice subjected to BCAS revealed fibre disarrangement, vacuoles and focal demyelination following BCAS. (B) Astrocytes, stained by an anti-GFAP antibody, became reactive, as indicated by higher area coverage, larger cell diameters, and longer processes. (C) The number of Iba1-positive microglia increased within the corpus callosum. (D) The signal intensity of axons stained with the pan-axonal marker SMI312 decreased after BCAS, indicative of axonal damage. (E) The number of NeuN-positive neuronal cell bodies was comparable in both groups. Scale bars, 100 μm. (PDF 7114 kb) [file 40478_2016_350_MOESM2_ESM.pdf]

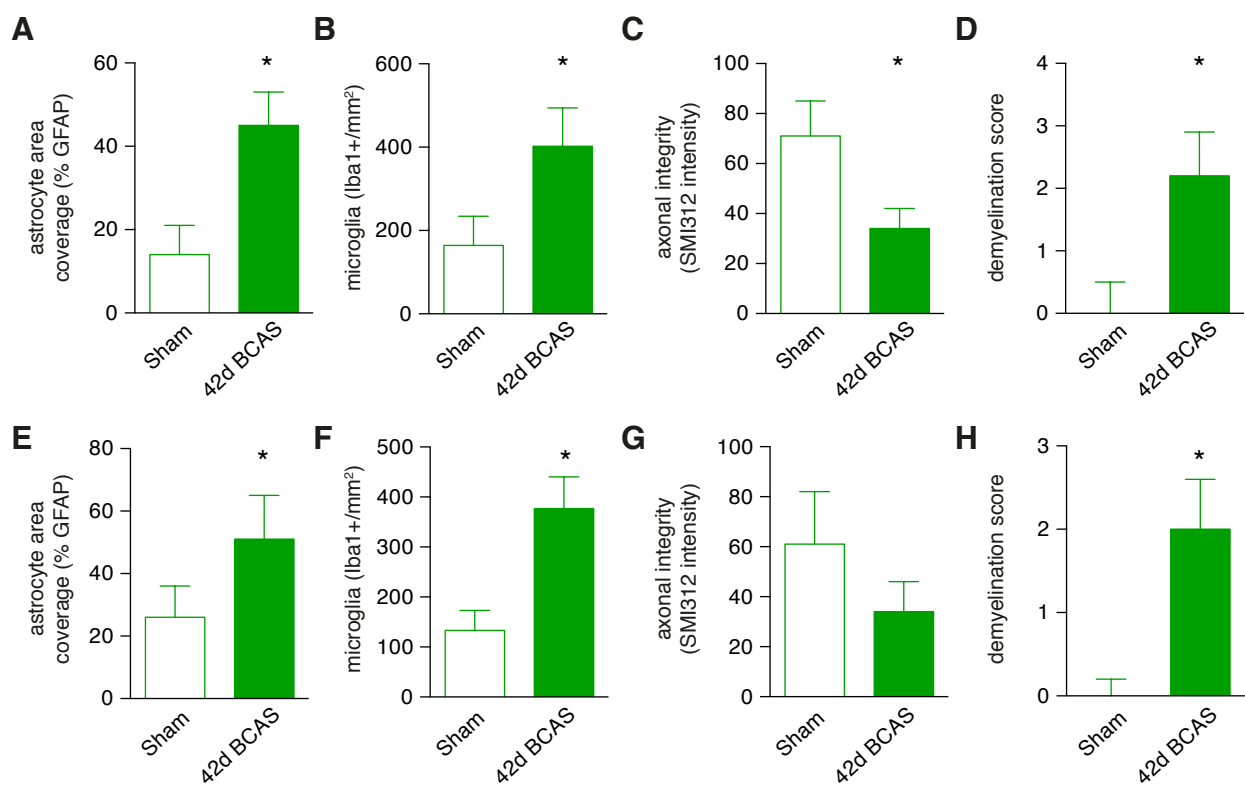

Supplement: Additional file 3: Figure S3. — Histological changes in the internal and external capsule. Reactive astrogliosis (A, E), microgliosis (B, F), axonal degeneration (C, G) and demyelination (D, H) in sham and BCAS animals in the internal capsule (upper row) and external capsule (lower row). (PDF 118 kb) [file 40478_2016_350_MOESM3_ESM.pdf]

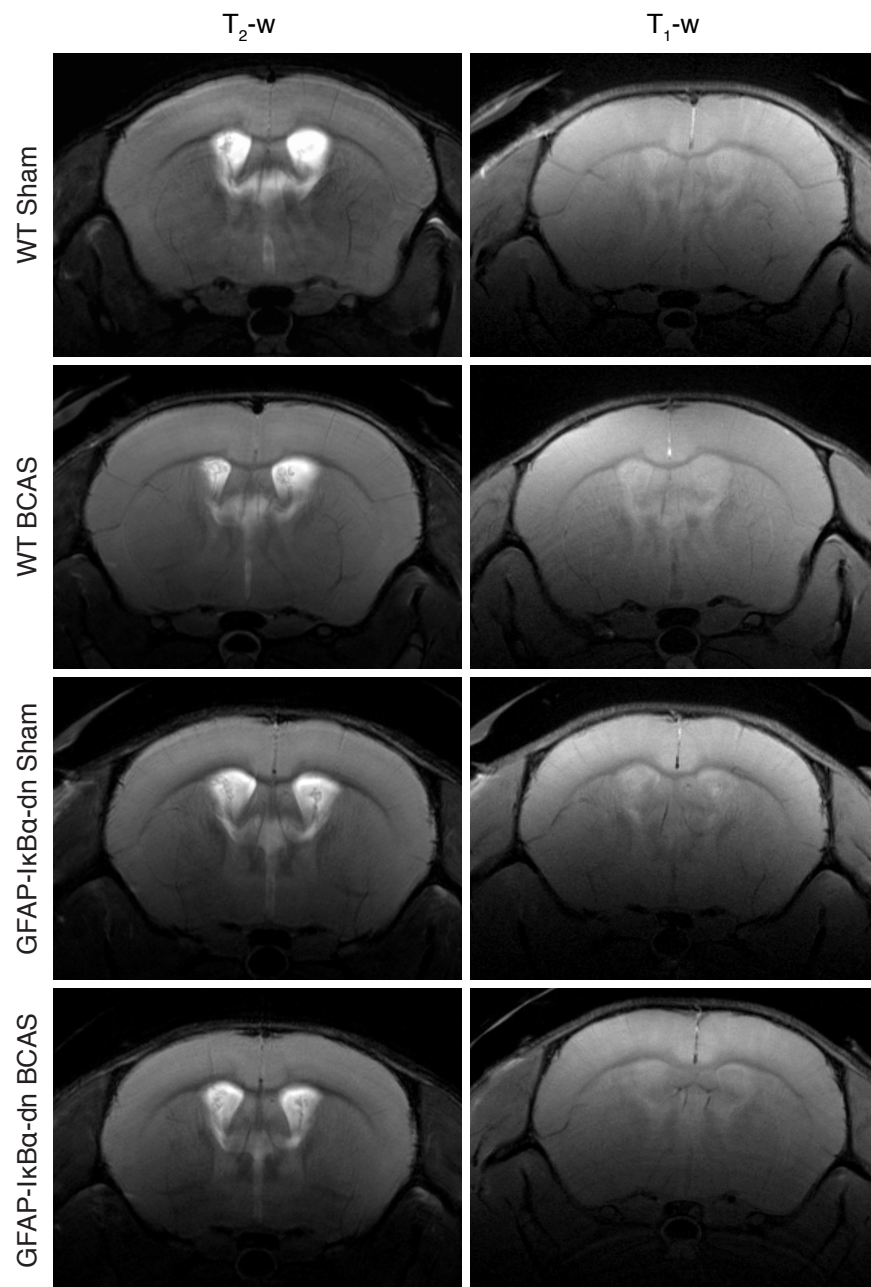

Supplement: Additional file 5: Figure S5. — T1- and T2-weighted images show no changes following BCAS. Representative examples of T1- and T2-weighted coronal images in wildtype or GFAP-IkBα-dn mice subjected to BCAS or sham surgery, respectively. These images show that grey and white matter structures are devoid of pathological signal changes. (PDF 2360 kb) [file 40478_2016_350_MOESM5_ESM.pdf]
