# Supplementary material for: Astroglial NF-kB contributes to white matter damage and cognitive impairment in a mouse model of vascular dementia
Source: Acta Neuropathol Commun. 2016 Aug 4;4:76. doi: 10.1186/s40478-016-0350-3 (PMC4973061; doi:10.1186/s40478-016-0350-3)
Supplement: Additional file 4: Figure S4. — Fractional anisotropy maps. Representative examples of fractional anisotropy maps in two coronal imaging planes (relative to Bregma) in wildtype or GFAP-IkBα-dn mice subjected to BCAS or sham surgery, respectively. (PDF 6689 kb) [file 40478_2016_350_MOESM4_ESM.pdf]

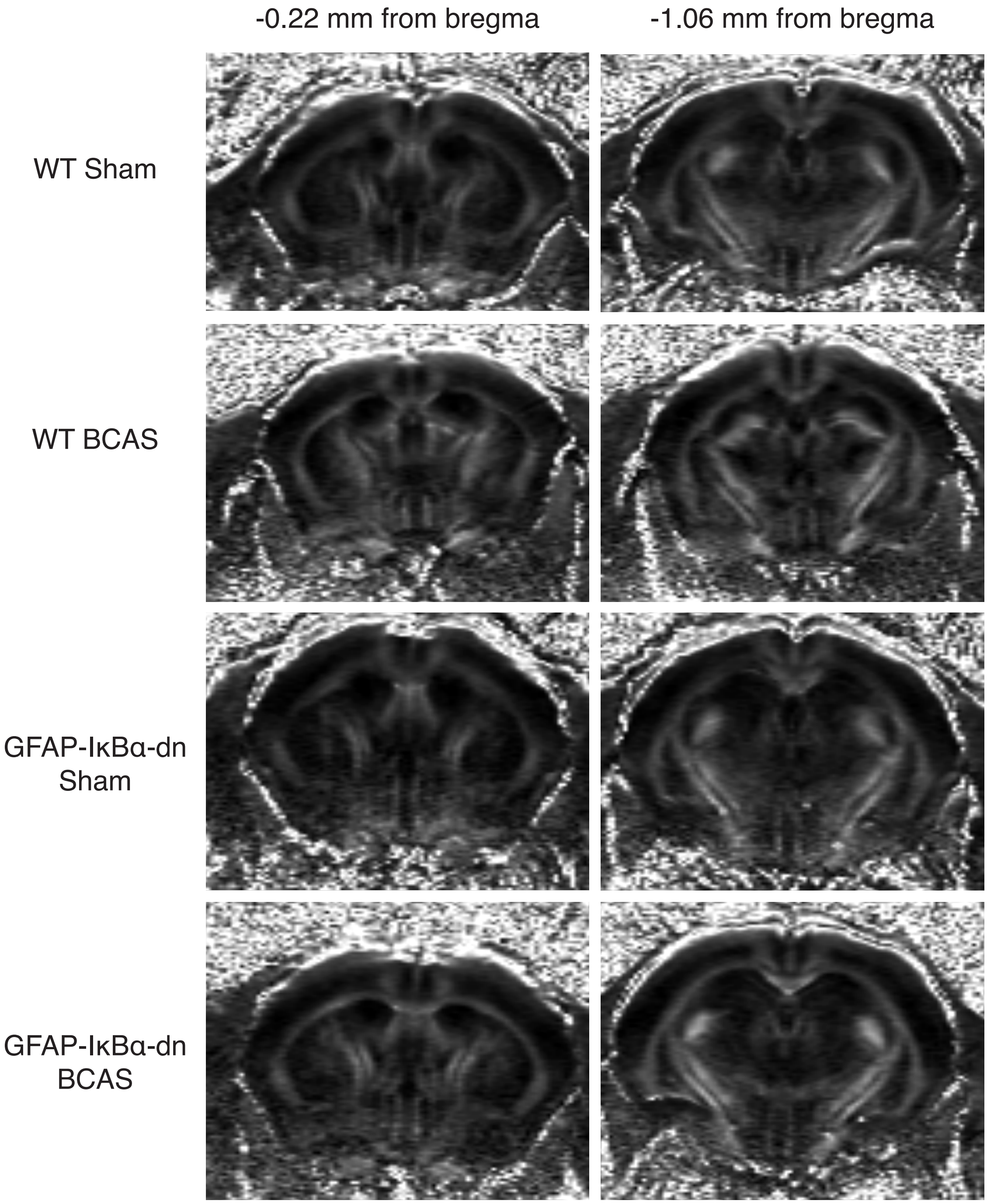
-0.22 mm from bregma
-1.06 mm from bregma
WT Sham
WT BCAS
GFAP-IκBα-dn Sham
GFAP-IκBα-dn BCAS
